# Morphologic diversity of the epididymis in orchiectomy specimens: a multi-institutional study

**DOI:** 10.1007/s00428-025-04390-1

**Published:** 2026-01-13

**Authors:** Busra Yaprak Bayrak, Ganime Coban, Murat Oktay, Fatma Aksoy Khurami, Deniz Baycelebi, Rabia Aktemur, Melike Karakuş Yılmaz, Fadime Eda Gokalp Satıcı, Merve Meryem Kiran, Yazgi Koy, Kemal Kösemehmetoğlu, Juan Sigala Lozano, Asli Noyan, Taha Cumhan Savli, Neşe Yeldir, Yasemin Yuyucu Karabulut, Busra Ozbek, Levent Trabzonlu, Mahmut Akgul

**Affiliations:** 1 Department of Pathology, School of Medicine, Faculty of Medicine, Kocaeli University, Kocaeli, 41,380 Turkey; 2 Department of Pathology, Faculty of Medicine, Bezmialem Vakıf University, Istanbul, Turkey; 3 Department of Pathology, Memorial Hospitals Group, Istanbul, Turkey; 4 Department of Pathology, Faculty of Medicine, Süleyman Demirel University, Isparta, Turkey; 5 Department of Pathology, Faculty of Medicine, Ondokuz Mayıs University, Samsun, Turkey; 6 Department of Pathology, Faculty of Medicine, Mersin University, Mersin, Turkey; 7 Department of Pathology, Ankara Bilkent State Hospital, Ankara, Turkey; 8 Department of Pathology, Health Sciences University Kocaeli Derince Training and Research Hospital, Kocaeli, Turkey; 9 Department of Pathology, Faculty of Medicine, Hacettepe University, Ankara, Turkey; 10 Department of Pathology, University of Illinois Chicago, Chicago, IL USA; 11 Department of Pathology, Faculty of Medicine, Istanbul Medipol University, Istanbul, Turkey; 12 Department of Pathology, Brigham and Women’s Hospital, Boston, USA; 13 Department of Pathology, Atlas University School of Medicine, Istanbul, Turkey

**Keywords:** Epididymis, Histopathology, Non-neoplastic morphology, Orchiectomy, Testicular pathology

## Abstract

The epididymis frequently exhibits a broad spectrum of non-neoplastic epithelial and stromal alterations that may mimic neoplastic or obstructive processes in orchiectomy specimens. Existing data are mostly derived from single-institution series. This multi-institutional study aimed to provide a comprehensive, contemporary, multi-institutional analysis of the prevalence, spectrum, and clinicopathological associations of epididymal morphological variations in a large orchiectomy cohort. This retrospective study included 1,528 orchiectomy specimens from multiple academic centers. All hematoxylin and eosin–stained slides containing epididymal tissue were systematically reviewed using a standardized protocol. Morphological features assessed included atrophy, intranuclear inclusions, lipofuscin pigment, cribriform hyperplasia, Paneth cell–like metaplasia, nuclear atypia, clear cell change, smooth-muscle proliferation, vascular and duct ectasia, myxoid change, calcification, hematoma, and inflammation. Associations with underlying testicular pathologies were analyzed statistically. 66% (1004/1528) were performed for testicular neoplasms, which were predominantly germ cell tumors derived from germ cell neoplasia in situ (87.5%, 878/1004). The most common epididymal alterations were lipofuscin pigment (49.9%, 762/1528), intranuclear inclusions (40.3%, 616/1528), atrophy (35.4%, 541/1528), and duct ectasia (35.3%, 539/1528). Non-tumoral cases more frequently exhibited atrophy (58.4%, 306/524 vs. 23.4%, 235/1004), duct ectasia (45.2%, 237/524 vs. 30.1%, 302/1004), inflammation (21.9%, 115/524 vs. 2.7%, 27/1004), and hematoma (5.9%, 31/524 vs. 0.2%, 2/1004) (p < 0.0001 for all). Tumoral cases showed higher rates of cribriform hyperplasia (28.5%, 286/1004 vs. 16.4%, 86/524), Paneth cell–like metaplasia (12.4%, 124/1004 vs. 1.9%, 10/524), nuclear atypia (21.9%, 220/1004 vs. 17.2%, 90/524), and clear cell change (21.7%, 218/1004 vs. 14.3%, 75/524) (all p ≤ 0.03). Several features, including atrophy, lipofuscin pigment, cribriform hyperplasia, clear cell change, and calcification, showed significant variation across tumor subtypes. Non-neoplastic epithelial and stromal alterations of the epididymis are common and histologically diverse, often co-occurring and varying by underlying testicular pathology. Awareness of these patterns is essential to avoid misinterpretation, especially in oncologic settings. This study provides the largest contemporary dataset to date, offering a robust histopathological framework for epididymal assessment in routine surgical pathology practice.

## Introduction

The epididymis is a highly specialized segment of the male reproductive tract, responsible for sperm maturation, storage, and transport. Its distinct anatomic regions exhibit unique epithelial architectures and functions, contributing to a finely regulated luminal microenvironment essential for post-testicular sperm differentiation [1, 2]. Despite its routine inclusion in orchiectomy specimens, histopathological evaluation of the epididymis has traditionally received less attention than that of the testis. In daily diagnostic practice, pathologists frequently encounter a variety of epithelial and stromal alterations—including intranuclear inclusions, lipofuscin pigment deposition, cribriform epithelial proliferations, Paneth cell–like granular changes, and nuclear atypia—that may mimic neoplastic or obstructive processes if not properly recognized [1–4].

A wide spectrum of non-neoplastic morphologic variations has been described in the epididymis, many of which may closely simulate pathologic entities if not carefully interpreted [1–5]. These alterations encompass epithelial and stromal changes such as intranuclear inclusions, lipofuscin pigment accumulation, cribriform proliferations, Paneth cell–like granular metaplasia, and focal nuclear atypia. Cribriform epithelial change, frequently observed in association with testicular atrophy, may mimic intraductal neoplasia but typically lacks significant cytological atypia or mitotic activity [1, 3]. Paneth cell–like changes, historically considered markers of obstruction, have been shown to represent lysosomal accumulations within the supranuclear cytoplasm and may occur independently of mechanical blockage [2]. Nuclear atypia and intranuclear inclusions, reminiscent of seminal vesicle epithelium, may appear striking but usually reflect degenerative phenomena without proliferative potential [1, 4]. Although the histologic spectrum of these findings has been well characterized, existing data are largely derived from single-institution cohorts or limited series, and comprehensive contemporary analyses remain scarce.

In the context of evolving clinical practices and increasing detection of early-stage testicular lesions, establishing robust reference data for epididymal morphology has become particularly relevant. The present multi-institutional study, encompassing a large contemporary orchiectomy cohort from nearly ten academic centers, systematically evaluated the prevalence, spectrum, and patterns of epididymal histologic variations and explores their associations with underlying testicular pathology. By integrating data from multiple institutions, this work provided the most extensive analysis to date, aiming to enhance diagnostic accuracy, reduce interpretative variability, and offered a modern histopathological framework for the assessment of epididymal morphology in surgical pathology practice.

## Materials and methods

### Patient selection

This retrospective, multi-institutional study was conducted on orchiectomy specimens collected from multiple academic centers over a defined study period. All available hematoxylin and eosin (H&E)-stained slides containing representative sections of the epididymis were retrieved from pathology archives and systematically reviewed by experienced pathologists. Bilateral specimens were assessed separately. Patients were included if epididymal tissue was well preserved and suitable for histological evaluation. Cases showing direct epididymal invasion by tumor were excluded, as such infiltration distorts the native epididymal architecture and precludes reliable assessment of non-neoplastic epididymal morphology.

Underlying testicular pathologies were classified into tumoral and non-tumoral categories based on histopathological review of the original slides and reports. Tumoral lesions included germ cell tumors, sex cord–stromal tumors, mesenchymal and adnexal tumors, hematolymphoid neoplasms, and metastases; non-tumoral lesions comprised atrophy, inflammation, cryptorchidism, trauma, and cystic lesions.

For demographic analyses, cases were stratified into pediatric (< 18 years) and adult (≥ 18 years) groups, consistent with standard clinical and epidemiologic conventions.

Ethical approval for this study was obtained from the Local Non-Interventional Research Ethics Committee (Decision No: KU GOKAEK-2025/22/38; Project No: 2025/572).

### Clinical data

Demographic and clinicopathological data, including patient age and the underlying testicular diagnosis, were collected from institutional medical records and pathology reports. All data were anonymized prior to analysis.

### Histological evaluation

**Fig. 1:**
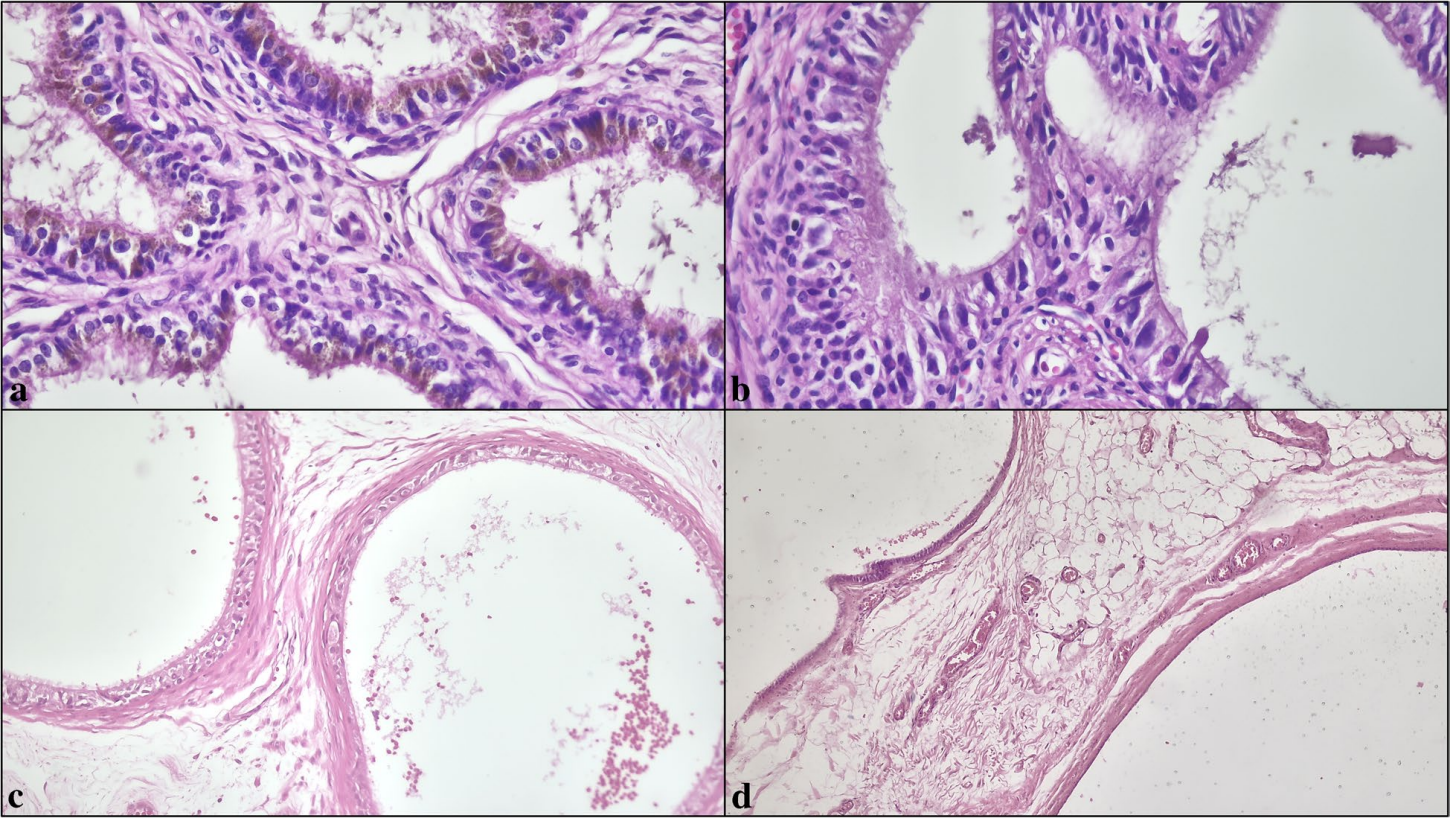
Representative epithelial alterations of the epididymis. (**a**) Lipofuscin pigment represented by coarse golden-brown cytoplasmic granules. (**b**) Intranuclear eosinophilic inclusions. (**c**) Atrophy characterized by thickened basement membranes and diminished epithelial complexity. (**d**) Duct ectasia with luminal distention (H&E, × 200–400)

**Fig. 2:**
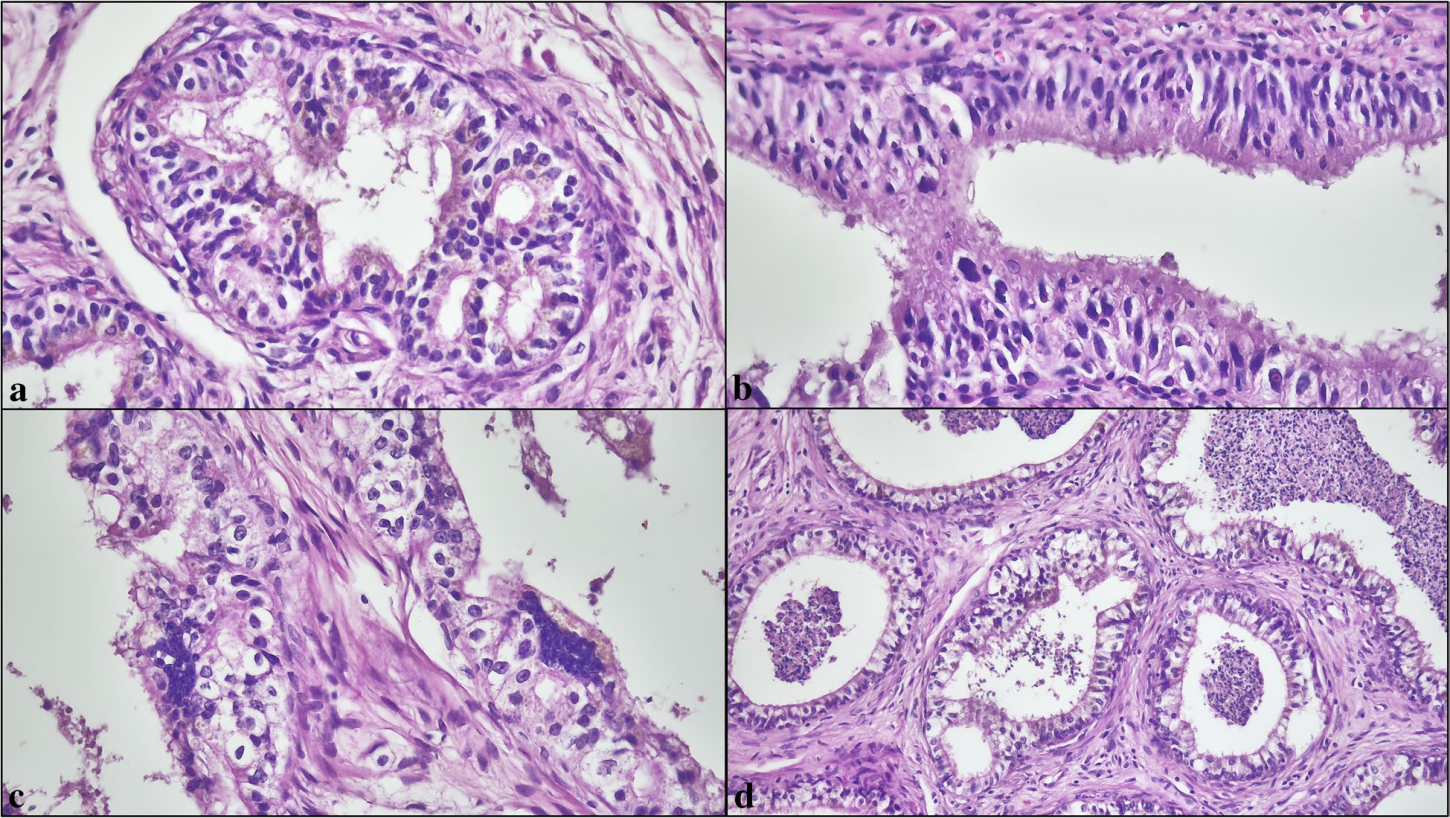
(**a**) Cribriform epithelial hyperplasia lacking cytologic atypia or mitotic activity. (**b**) Nuclear atypia with focal nuclear enlargement and irregular contours. (**c**) Multinucleated giant cell formations within the epithelium. (**d**) Clear cell change composed of pale-staining epithelial cells (H&E, × 200–400)

**Fig. 3:**
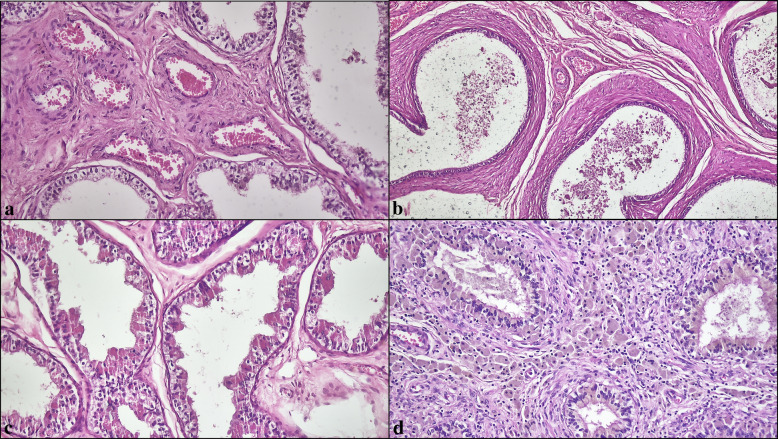
(**a**) Vascular ectasia with dilated stromal vessels. (**b**) Concentric smooth muscle proliferations surrounding ducts. (**c**) Paneth cell–like metaplasia showing supranuclear eosinophilic granules. (**d**) Inflammation involving periductal and interstitial compartments (H&E, × 100–200)

All H&E slides were evaluated using light microscopy. A standardized protocol was applied to document the presence or absence of predefined non-neoplastic morphologic variations in the epididymis. The features assessed included testicular atrophy, intranuclear eosinophilic inclusions, lipofuscin pigment accumulation, cribriform epithelial hyperplasia, Paneth cell–like metaplasia, nuclear atypia, clear cell change, smooth-muscle proliferations around the ducts, vascular ectasia, duct ectasia, myxoid stromal change, stromal or ductal calcification, hematoma, and inflammation (Figs. 1–3).

Atrophy was defined by thickened basement membranes, reduced or absent spermatogenesis, and interstitial fibrosis with or without Leydig cell prominence. Intranuclear inclusions were characterized by round, densely eosinophilic bodies within epithelial nuclei, and lipofuscin pigment by fine golden-brown cytoplasmic granules. Cribriform hyperplasia referred to sieve-like epithelial proliferations lacking cytologic atypia or mitotic activity, whereas Paneth cell–like metaplasia consisted of brightly eosinophilic supranuclear cytoplasmic granules. Nuclear atypia encompassed focal epithelial nuclear enlargement with irregular contours and chromatin clumping without mitotic figures. Clear cell changes denoted pale-staining epithelial cells typically observed in the cauda. Smooth-muscle proliferations represented periductal concentric thickening. Vascular and duct ectasia referred to dilatation of stromal vessels and ducts, respectively. Myxoid change indicated loose, basophilic stromal alterations; calcifications were identified as granular or lamellated basophilic deposits; hematoma indicated extravasated erythrocytes or organized blood collections; and inflammation referred to acute or chronic inflammatory infiltrates in periductal or stromal areas. Multiple features could be recorded in the same patient.

### Statistical analysis

All statistical analyses were performed using GraphPad InStat software (GraphPad Software Inc., San Diego, CA, USA). Data distribution was assessed for normality using the Kolmogorov–Smirnov test. The frequency and distribution of each histological feature were documented and stratified by underlying testicular pathology. Associations between morphologic features and clinicopathological variables were analyzed using Chi-square or Fisher’s exact tests for categorical variables and t-tests or Mann–Whitney U tests for continuous variables. Correlations between morphologic features were assessed using Spearman’s rank correlation coefficient. A *p* value < 0.05 was considered statistically significant.

## Results

The mean age of 1528 patients were 36 ± 20 years (median: 32; range: 0–96). 11.1% of patients (*n* = 170) were pediatrics while 88.9% of them were adults (*n* = 1358). Intra-testicular neoplasms accounted for 66% of cases (*n* = 1004), while non-tumoral pathologies represented 34% (*n* = 524). In the pediatric population, the majority of cases were non-tumoral (79%) compared with 21% tumoral pathologies. Conversely, in adult patients, tumoral lesions were more frequent (71%) than non-tumoral lesions (29%).

Among the testicular tumors, the vast majority were germ cell tumors derived from germ cell neoplasia in situ (GCNIS), comprising 878 cases (87.5%) (Table 1). Germ cell tumors unrelated to GCNIS were rare, observed in only 12 cases (1.2%). Sex cord-stromal tumors accounted for 42 cases (4.2%), while hematolymphoid tumors represented 32 cases (3.2%). Mesenchymal tumors were identified in 21 cases (2.1%), and tumors of the testicular adnexa in 14 cases (1.4%). Metastatic tumors involving the testis were extremely uncommon, with only 5 cases (0.5%). These metastatic tumors originated from colorectal adenocarcinoma (n = 1), Wilms tumor (*n* = 1), and prostatic adenocarcinoma (*n* = 3). All metastatic deposits were confined to the testicular parenchyma without epididymal involvement.

**Table 1:** Distribution of Testicular Tumor Subtypes

Tumor Subtypes	*n*	%
Germ cell tumors derived from germ cell neoplasia in situ	878	87.5
Germ cell tumors unrelated to germ cell neoplasia in situ	12	1.2
Sex cord stromal tumors of the testis	42	4.2
Hematolymphoid tumors	32	3.2
Mesenchymal tumors	21	2.1
Metastasis to the testis	5	0.5
Tumors of the testicular adnexa	14	1.4

Among malignant germ cell tumors, rete testis invasion was identified in approximately 36% of cases. None of the malignant germ cell tumors demonstrated epididymal invasion, and all epididymal changes observed in this study represented non-neoplastic alterations rather than tumor infiltration.

The primary localization of documented morphological changes was the ductuli efferentes in 237 patients (15.5%), the ductus epididymis in 415 patients (27.2%), and both sites in 876 patients (57.3%). In the full cohort of epididymal pathologies (*N* = 1528), the most prevalent alterations were lipofuscin pigment (762/1528, 49.9%) (Fig. 1a), intranuclear inclusions (616/1528, 40.3%) (Fig. 1b), atrophy (541/1528, 35.4%) (Fig. 1c), and duct ectasia (539/1528, 35.3%) (Fig. 1d). Cribriform hyperplasia (372/1528, 24.3%) (Fig. 2a), nuclear atypia (310/1528, 20.3%) (Fig. 2b, c), clear cell change (293/1528, 19.2%) (Fig. 2d), vascular ectasia (272/1528, 17.8%) (Fig. 3a), and smooth-muscle proliferations around ducts (198/1528, 13.0%) (Fig. 3b) were less frequent, whereas Paneth cell–like metaplasia (134/1528, 8.8%) (Fig. 3c), inflammation (142/1528, 9.3%) (Fig. 3d), hematoma (33/1528, 2.2%), calcification (14/1528, 0.9%), and myxoid change (2/1528, 0.1%) were uncommon (Table 2).

**Table 2:** Distribution of Morphological Features by Pathology Type of Epididymis

Features N (%)	Total (*N* = 1528)	Non-tumoral (*N* = 524)	Tumoral (*N* = 1004)	*P* value
Lipofuscin Pigment	762 (49.9)	252 (48.1%)	510 (50.8%)	0.3154
Intranuclear Inclusions	616 (40.3)	201 (38.4%)	415 (41.3%)	0.2603
Atrophy	541 (35.4)	306 (58.4%)	235 (23.4%)	** < 0.0001**
Duct Ectasia	539 (35.3)	237 (45.2%)	302 (30.1%)	** < 0.0001**
Cribriform Hyperplasia	372 (24.3)	86 (16.4%)	286 (28.5%)	** < 0.0001**
Nuclear Atypia	310 (20.3)	90 (17.2%)	220 (21.9%)	**0.0288**
Clear cell change	293 (19.2)	75 (14.3%)	218 (21.7%)	**0.0005**
Vascular Ectasia	272 (17.8)	108 (20.6%)	164 (16.3%)	**0.0381**
Smooth muscle proliferations	198 (12.9)	85 (16.2%)	113 (11.3%)	**0.0061**
Inflammation	142 (9.3)	115 (21.9%)	27 (2.7%)	** < 0.0001**
Paneth Cell-like Metaplasia	134 (8.8)	10 (1.9%)	124 (12.4%)	** < 0.0001**
Hematoma	33 (2.2)	31 (5.9%)	2 (0.2%)	** < 0.0001**
Calcification	14 (0.9)	9 (1.7%)	5 (0.5%)	**0.0175**
Myxoid Change	2 (0.1)	0 (0.0%)	2 (0.2%)	0.3066

When stratified by diagnostic category, non-tumoral lesions (*N* = 524) more often showed atrophy (306/524, 58.4% vs. 235/1004, 23.4%), duct ectasia (237/524, 45.2% vs. 302/1004, 30.1%), inflammation (115/524, 21.9% vs. 27/1004, 2.7%), hematoma (31/524, 5.9% vs. 2/1004, 0.2%), calcification (9/524, 1.7% vs. 5/1004, 0.5%), vascular ectasia (108/524, 20.6% vs. 164/1004, 16.3%), and thick and concentric smooth muscle proliferations around the ducts (85/524, 16.2% vs. 113/1004, 11.3%). In contrast, tumoral lesions (*N* = 1004) more frequently exhibited cribriform hyperplasia (286/1004, 28.5% vs. 86/524, 16.4%), Paneth cell–like metaplasia (124/1004, 12.3% vs. 10/524, 1.9%), nuclear atypia (220/1004, 21.9% vs. 90/524, 17.2%), and clear cell change (218/1004, 21.7% vs. 75/524, 14.3%). The frequencies of intranuclear inclusions (41.3% vs. 38.4%) and lipofuscin pigment (50.8% vs. 48.1%) were comparable between tumoral and non-tumoral groups (Table 2).

Across tumor subtypes, several morphological features varied significantly. Atrophy differed by subtype (*p* = 0.044), peaking in metastases to the testis (4/5, 80%) and GCNIS-unrelated germ cell tumors (5/12, 41.7%), versus GCNIS-derived tumors (196/878, 22.3%) (Table 3). Lipofuscin pigment also varied (*p* = 0.028), being most frequent in mesenchymal tumors (16/21, 76.2%) and tumors of the testicular adnexa (10/14, 71.4%), with high rates in hematolymphoid tumors (20/32, 62.5%), compared with GCNIS-derived tumors (441/878, 50.2%). Cribriform hyperplasia showed subtype enrichment (*p* = 0.006), common in GCNIS-derived tumors (266/878, 30.3%) and adnexal tumors (4/14, 28.6%), present in sex cord-stromal tumors (11/42, 26.2%), but absent in GCNIS-unrelated tumors (0/12) and metastases (0/5). Clear-cell change differed across groups (*p* = 0.020), being most frequent in GCNIS-derived (206/878, 23.5%) and adnexal tumors (3/14, 21.4%), lower in sex cord-stromal (5/42, 11.9%) and hematolymphoid tumors (2/32, 6.3%), and absent in GCNIS-unrelated and metastatic tumors. Calcification was rare overall but showed a subtype signal (*p* = 0.012), occurring mainly in sex cord-stromal tumors (2/42, 4.8%) and scarcely in GCNIS-derived tumors (3/878, 0.3%).

**Table 3:** Distribution of Morphological Features by Pathology Type of Epididymis

Features *N* (%)	Germ cell tumors derived from germ cell neoplasia in situ (*n* = 878)	Germ cell tumors unrelated to germ cell neoplasia in situ (*n* = 12)	Sex cord stromal tumors of the testis (*n* = 42)	Hematolymphoid tumors (*n* = 32)	Mesenchymal tumors (*n* = 21)	Metastasis to the testis (*n* = 5)	Tumors of the testicular adnexa (*n* = 14)	*P* value
Lipofuscin Pigment	441 (50.2)	3 (25)	18 (42.9)	20 (62.5)	16 (76.2)	2 (40)	10 (71.4)	**0.0279**
Intranuclear Inclusions	359 (40.9)	2 (16.7)	24 (57.1)	10 (31.3)	10 (47.6)	2 (40)	8 (57.1)	0.1036
Atrophy	196 (22.3)	5 (41.7)	12 (28.6)	8 (25)	6 (28.6)	4 (80)	4 (28.6)	**0.0442**
Duct Ectasia	270 (30.8)	0 (0)	15 (35.7)	5 (15.6)	6 (28.6)	1 (20)	5 (35.7)	0.1407
Cribriform Hyperplasia	266 (30.3)	0 (0)	11 (26.2)	2 (6.3)	3 (14.3)	0 (0)	4 (28.6)	**0.0059**
Nuclear Atypia	198 (22.6)	1 (8.3)	10 (23.8)	3 (9.4)	5 (23.8)	0 (0)	3 (21.4)	0.4255
Clear cell change	206 (23.5)	0 (0)	5 (11.9)	2 (6.3)	2 (9.5)	0 (0)	3 (21.4)	**0.0202**
Vascular Ectasia	135 (15.4)	4 (33.3)	14 (33.3)	5 (15.6)	3 (14.3)	1 (20)	2 (14.3)	0.0581
Smooth muscle proliferations	95 (10.8)	3 (25)	8 (19.1)	3 (9.4)	1 (4.8)	1 (20)	2 (14.3)	0.3695
Inflammation	22 (2.5)	1 (8.3)	2 (4.8)	0 (0)	2 (9.5)	0 (0)	0 (0)	0.2837
Paneth Cell-like Metaplasia	119 (13.6)	0 (0)	1 (2.4)	4 (12.5)	0 (0)	0 (0)	0 (0)	0.0544
Hematoma	2 (0.2)	0 (0)	0 (0)	0 (0)	0 (0)	0 (0)	0 (0)	0.9996
Calcification	3 (0.3)	0 (0)	2 (4.8)	0 (0)	0 (0)	0 (0)	0 (0)	**0.0124**
Myxoid Change	2 (0.2)	0 (0)	0 (0)	0 (0)	0 (0)	0 (0)	0 (0)	0.9996

Other features did not differ significantly by subtype: intranuclear inclusions (*p* = 0.104), Paneth cell-like metaplasia (*p* = 0.054, showing a non-significant trend toward higher frequency in tumoral cases), nuclear atypia (*p* = 0.426), smooth-muscle proliferations (*p* = 0.370), vascular ectasia (*p* = 0.058, demonstrating a non-significant trend toward higher rates in non-tumoral cases), duct ectasia (*p* = 0.141), myxoid change (*p* = 0.999), hematoma (*p* = 0.999), and inflammation (*p* = 0.284) (Table 3). Taken together, atrophy, lipofuscin pigment, cribriform hyperplasia, clear-cell change, and (rare) calcification show the most discriminative distributions across epididymal tumor subtypes.

## Discussion

This large multi-institutional study provides the most comprehensive contemporary analysis of non-neoplastic epididymal morphology in orchiectomy specimens to date. By evaluating more than 1,500 patients across multiple centers, we systematically characterized a broad spectrum of epithelial and stromal variations and correlated these features with underlying testicular pathology. Our findings confirm that these morphologic alterations are not uncommon and often coexist, underscoring the importance of recognizing their histologic patterns to avoid diagnostic pitfalls in routine surgical pathology practice.

The spectrum and frequency of morphologic variations identified in our cohort parallel those described in previous single-institution studies, albeit with notable differences in prevalence. Earlier investigations have documented intranuclear inclusions in approximately 70% of cases, lipofuscin pigment in about one-third, cribriform hyperplasia in 40%, Paneth cell–like metaplasia in 8%, and nuclear atypia in 14% [1, 5]. Our multi-institutional analysis corroborated these frequencies, while also providing more granular data on less commonly reported stromal alterations such as smooth muscle proliferations, myxoid change, and vascular ectasia, which have received little attention in the literature [1, 3]. Importantly, multiple epithelial and stromal features frequently coexisted in individual specimens, reflecting the complex histologic milieu of the epididymis in diseased testes.

Several morphologic changes merit particular attention because of their potential to mimic neoplastic or obstructive processes. Cribriform hyperplasia is a well-recognized pseudo neoplastic pattern that can closely resemble intraductal proliferations of malignant tumors but typically lacks cytologic atypia or mitotic activity. Previous reports have associated this change with testicular atrophy and rete testis adenomatous hyperplasia [4, 5]. In our series, cribriform hyperplasia was common in both tumoral and non-tumoral settings. Similarly, Paneth cell–like changes—traditionally considered markers of obstruction—are now understood to represent lysosomal accumulations rather than true secretory metaplasia [2]. We observed these changes across a range of clinical settings, including cases without morphologic evidence of obstruction, consistent with prior immunohistochemical studies demonstrating their lysosomal nature rather than phospholipase A2 expression [2, 6, 7].

When non-tumoral testicular lesions are set aside, it becomes evident that many germ cell tumors in our series involved the rete testis. This observation aligns with the obstruction-based mechanism proposed by Potterveld et al. in their multi-institutional study, in which reactive hyperplasia and hyaline globule formation in the rete testis were shown to arise not only from direct tumor invasion but also as secondary changes related to tumor-induced outflow obstruction [8]. Potterveld et al. further demonstrated that similar obstruction-related alterations can develop within epididymal structures, reporting Paneth cell–like metaplasia in 13% of efferent ductule/epididymal samples [8]. In our cohort, Paneth cell–like metaplasia was observed in 8.8% (134/1528) of cases, a frequency that closely parallels the rates described in the literature. Taken together, these similarities suggest that some of the non-neoplastic epididymal changes identified in our study may, at least in a subset of cases, represent reactive adaptive responses secondary to tumor-related obstruction of testicular outflow pathways.

Beyond their biological significance, a central practical contribution of this study is the delineation of specific morphologic variations that may mimic true neoplastic processes and thereby pose diagnostic or staging challenges. Given the high volume of orchiectomy specimens evaluated in routine practice, distinguishing benign epididymal alterations from entities such as epididymal adenoma, papillary cystadenoma, metastatic tumors, or direct extension of germ cell tumors is essential. Cribriform hyperplasia, for example, may be misinterpreted as intraductal carcinoma when encountered adjacent to a testicular tumor. Diagnostic separation relies on several reproducible features, including preservation of ductal contours, uniform cellularity, bland nuclei, and absence of mitotic figures.

Similarly, Paneth cell–like metaplasia may simulate yolk sac tumor differentiation or metastatic clear cell tumors due to its prominent eosinophilic supranuclear granularity. Awareness of the lysosomal, non-secretory nature of these granules—as well as their frequent occurrence in non-tumoral settings—helps avoid unnecessary immunohistochemical workup or overdiagnosis. Nuclear atypia, when focal and degenerative, may mimic early dysplastic change or tumoral epithelial involvement, but careful evaluation reveals smooth nuclear membranes, lack of stratification, and absence of proliferation.

Stromal alterations also have staging implications. Concentric periductal smooth muscle hyperplasia may resemble desmoplastic stromal reaction or early extratesticular invasion if not carefully distinguished from true infiltrative tumor patterns. Marked duct ectasia with intraluminal eosinophilic debris may imitate lymphovascular invasion, particularly in fragmented orchiectomy specimens, leading to potential over-staging. Atrophic, compressed epididymal tubules adjacent to large mass-forming tumors may falsely suggest epididymal invasion and erroneous assignment of pT2 disease. These distinctions are critical because overstaging may lead to overtreatment.

Intranuclear inclusions and nuclear atypia represent additional sources of potential confusion. These degenerative alterations, reminiscent of seminal vesicle epithelium, may appear alarming at low magnification but generally lack proliferative activity or clinical significance [1, 9]. Their high prevalence, particularly in older patients and in association with testicular atrophy, highlights the need for awareness among pathologists to prevent overdiagnosis. Lipofuscin pigment accumulation, predominantly in the efferent ductules, was also frequently encountered and, as reported previously, often accompanied obstructive changes, although it can occur as an age-related phenomenon [1, 6].

Beyond epithelial alterations, our study also systematically assessed stromal changes, which have been relatively underreported in the literature. Features such as periductal smooth muscle proliferations, vascular and duct ectasia, stromal myxoid change, and calcifications were documented and often coexisted with epithelial alterations. Recognizing these changes and their frequent overlap may assist in distinguishing non-neoplastic variations from pathologic processes such as inflammation, fibrosis, or tumor infiltration [3, 7, 10].

Taken together, these findings highlight that non-neoplastic epithelial and stromal variations of the epididymis are both common and histologically diverse, often occurring in complex combinations within the same specimen. In routine practice, unawareness of these patterns may lead to diagnostic overinterpretation, particularly in the context of tumor resections or small biopsy specimens. Establishing clear morphologic criteria and understanding their clinicopathologic context are therefore essential for accurate histopathological evaluation. Moreover, emerging molecular and single-cell studies are beginning to shed light on the functional heterogeneity of epididymal segments, which may ultimately refine the interpretation of these changes within their biological framework [11–15].

In conclusion, this multi-institutional analysis delineates the prevalence and spectrum of non-neoplastic morphologic variations of the epididymis and underscores their frequent association with testicular pathology. Awareness of these changes is essential to avoid misdiagnosis, particularly in the context of tumor resections and orchiectomy specimens. Our findings provide a modern histopathological framework for interpreting epididymal morphology and highlight the value of systematic evaluation in routine practice.

## Data Availability

All data generated or analyzed during this study are included in this published article. Data available on request due to privacy/ethical restrictions.
